# Supplementary material for: Identification and Validation of a Prognostic Signature Derived from the Cancer Stem Cells for Oral Squamous Cell Carcinoma
Source: Int J Mol Sci. 2024 Jan 14;25(2):1031. doi: 10.3390/ijms25021031 (PMC10816075; doi:10.3390/ijms25021031)
Supplement: Supplementary file 1 [file ijms-25-01031-s001.zip › ijms-2818386 Figure S1.pdf]

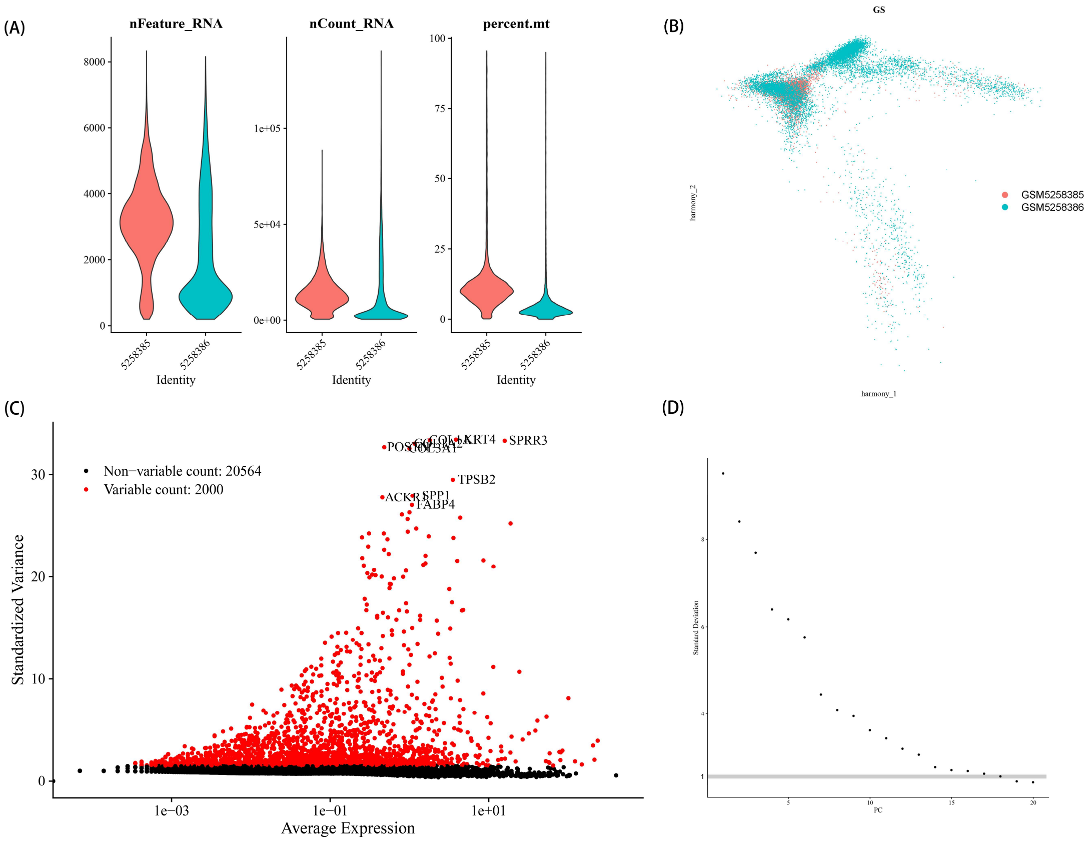

Figure S1. Data quality control and visualization. (**A**) The gene numbers, cell numbers, and the mitochondrial contents of samples. (**B**) Multiple PC populations with large differences. (**C**) Calculated and visualized HVGs (**D**)The scree plot of the results; Table S1: Identification of cancer stem cell-related genes
